# Dynamics of Word Production in the Transition from Adolescence to Adulthood

**DOI:** 10.1162/nol_a_00024

**Published:** 2020-11-01

**Authors:** Tanja Atanasova, Raphaël Fargier, Pascal Zesiger, Marina Laganaro

**Affiliations:** Faculty of Psychology and Educational Sciences, University of Geneva, Geneva, Switzerland; Laboratoire Parole et Langage, Aix-Marseille University, Marseille, France; Faculty of Psychology and Educational Sciences, University of Geneva, Geneva, Switzerland; Faculty of Psychology and Educational Sciences, University of Geneva, Geneva, Switzerland

**Keywords:** ERP, language production, adolescence, language development, topographic maps, electrophysiology

## Abstract

Changes in word production occur across the lifespan. Previous studies have shown electrophysiological, temporal, and functional differences between children and adults accompanying behavioral changes in picture-naming tasks (Laganaro, Tzieropoulos, Fraunfelder, & Zesiger, 2015). Thus, a shift toward adult-like processes in referential word production occurs somewhere between the ages of 13 and 20. Our aim was to investigate when and how children develop adult-like behavior and brain activation in word production. Toward this aim, performance and event-related potentials (ERP) in a referential word production task were recorded and compared for two groups of adolescents (aged 14 to 16 and 17 to 18), children (aged 10 to 13), and young adults (aged 20 to 30). Both groups of adolescents displayed adult-like production latencies, which were longer only for children, while accuracy was lower in the younger adolescents and in children, compared to adults. ERP waveform analysis and topographic pattern analysis revealed significant intergroup differences in key time-windows on stimulus-locked ERPs, both early (150–220 ms)—associated with pre-linguistic processes—and late (280–330 ms)—associated with lexical processes. The results indicate that brain activation underlying referential word production is completely adult-like in 17-year-old adolescents, whereas an intermediate pattern is still observed in adolescents aged 14 to 16 years old, although their production speed, but not their accuracy, is already adult-like.

## INTRODUCTION

The social aptitude of speaking is universal and unique to our species; converting prelinguistic concepts into articulated words and sentences within an ephemeral time period represents one of the major human-specific abilities. This ability is manifested from the moment children say their first word (i.e., around the twelfth month after birth), yet the accuracy and the speed of word production evolve during the lifespan, along with the size of the vocabulary. The lexicon grows from approximately 300 entries after one year of life (Dale & Fenson, 1996) to 10,000 by the age of six (Clark, 1993), and about 60,000 words in adulthood (Miller, 1996).

Compared to adults, school-aged children present reduced vocabulary, lower accuracy, and slower processing speed. Nonetheless, the same psycholinguistic variables, namely lexical frequency, concept familiarity, word age of acquisition (AoA), visual complexity of pictures, and word length, seem to predict both accuracy and production latencies in children (Cycowicz, Friedman, Rothstein, & Snodgrass, 1997), as well as in adults (D’Amico, Devescovi, & Bates, 2001). This suggests that the same encoding processes underlie single-word production in children and adults, regardless of the behavioral differences between the two groups. Such a claim is challenged by functional neuroimaging results showing that differences between children and adults are not limited to processing speed; in fact, some, but not all, mental processes underlying word production in picture-naming tasks seem to differ between children and adults (Laganaro, Tzieropoulos, Fraunfelder, & Zesiger, 2015). Electrophysiological event-related potentials (ERPs) of referential word production differ both qualitatively (i.e., different underlying neural generators) and quantitatively (i.e., different durations of similar processes) between school-aged children (7 to 8 and 10 to 12 years old) and young adults (Laganaro et al., 2015), leaving open the issue of *when* the brain mechanisms underlying word production become adult-like. Therefore, the present study intends to fill the gap between childhood and adulthood, by investigating the functional and temporal dynamics of changes in word production from childhood to adulthood, focusing on adolescence.

Most of the psycholinguistics models of speech production based on adult behavior agree that word production involves three main levels of processing, namely conceptual, lexical, and phonological-phonetic processes (Dell, 1986; Levelt, 1989). The dynamics of these mental processes have largely been investigated on populations of young adults, both behaviorally and with neuroimaging techniques. Indefrey and Levelt (2004; see also Indefrey, 2011) have framed precise spatial and temporal functional features of word production through a meta-analysis of previously published behavioral and event-related EEG/MEG studies using pictures to elicit utterance production. The authors proposed distinctive markers of lexical (∼200 ms after picture presentation) and phonological (∼450 ms after picture presentation) processing consistent with the abovementioned language production models. Other authors have proposed different dynamics, involving parallel activation of lexical and phonological information (Miozzo, Pulvermüller, & Hauk, 2014; Strijkers, Costa, & Pulvermuller, 2017), but empirical evidence is not yet conclusive in favor of one or the other model (Munding, Dubarry, & Alario, 2016).

In referential naming tasks, however, children and adults largely differ in accuracy and speed: Children display about 300 to 400 ms longer production latencies than adults (D’Amico et al., 2001). The similarity in the psycholinguistic predictors of referential naming may suggest that the same processes underlie word production in children and adults, and that only timing differs—that is, the same underlying processes are scaled in time and that the differences between adults’ and children’s speech production systems are quantitative rather than qualitative. This intuition has been challenged by some qualitative differences observed in neuroimaging studies directly comparing utterance production in children and adults. Laganaro et al. (2015) compared children to adults in a picture-naming task using global field pattern (topographic) ERP analyses to establish whether the differences in production speed were due to functional dissimilarity or to analogous processes merely shifted in time. The authors showed that the longer production latencies in 10- to 12-year-old children relative to adults were associated with both quantitative (temporal changes, same topographies shifted in time) and qualitative differences (functional changes, different topographies). In particular, qualitative functional differences were observed in early time-windows, around the P1 and N1 components and likely related to pre-linguistic processes. Other ERP studies investigating language production in children reported mainly temporal shifts in 12-year-old children relative to adults in a verb production task (Budd, Paulmann, Barry, & Clahsen, 2013) or enhanced amplitudes in children relative to adults in a picture-naming and word-reading task (Greenham & Stelmack, 2001). In terms of spatial resolution, functional magnetic resonance imaging (fMRI) comparing language production in children and adults showed fundamentally overlapping activations between the two groups, but also higher hemodynamic response in children (Brown et al., 2005), just as the opposite (Brown et al., 2005; Krishnan, Leech, Mercure, Lloyd-Fox, & Dick, 2014). Krishnan et al. (2014) suggested that differences in the activation patterns among children and adults underlay the qualitative changes in processes, affecting the delay and the strength of the spatial activation.

Hence, previous studies have shown clear behavioral changes between school-age children and adults, both in terms of accuracy and speed, whereas it is still unclear whether different brain processes in word production underpin such changes. Even more unclear is *when*, during development, performance and brain processes underlying word production become adult-like. Adolescence is likely the transition period when a shift toward adult-like processes occurs both in behavior and in brain processes. It is certainly well known that the brain itself undergoes massive changes during the teenage years. MRI studies have shown that the cerebral cortex seems to shrink during adolescence, when significant region-specific structural and functional changes are observed, leading to a unique pattern of brain responses and cognition (Dumontheil, 2016). Structural changes are detected in both grey and white matter, with grey matter volume reaching its peak during mid- to late childhood, decreasing during adolescence (Dumontheil, 2016). Cortical thinning is believed to be associated with underlying synaptic pruning (Alemán-Gómez et al., 2013); counterintuitively, a higher degree of pruning seems to be observed in highly used brain processes, which would allow associations requiring constant updating to have a larger palette of neuronal connections, linked with higher cognitive functions such as language (Riley et al., 2018). This phenomenon is predominantly detected in frontal and occipital cortices, as a consequence of sulcal widening, linked to cortical thinning, which is associated with gyral white matter expansion (Alemán-Gómez et al., 2013). White matter has been shown to increase linearly during the first two to three decades of life (Dumontheil, 2016). White matter’s growth probably reflects changes in myelination and axonal diameter (Alemán-Gómez et al., 2013), with white matter pathways showing progressive maturation during adolescence and consequently reflecting the changes in cognitive performance (Riley et al., 2018). These changes can also be detected with EEG.

In terms of EEG power, the elimination of active synapses and changes in white matter may be the reflection of the neural processes driving the age-related decreases in low frequencies (in absolute power measure) and increases in high frequencies (only in relative power; Goossens, Vercammen, Wouters, & van Wieringen, 2016). The whole process is, however, indicative of continuing development of the thalamo-cortical and cortico-cortical networks that generate EEG activity (Cragg et al., 2011). Axonal myelination and the integrity of white matter pathways have been suggested to underlie peak alpha frequency shifts (Miskovic et al., 2015). From early to late childhood, alpha-band electro-cortical connectivity becomes more integrated, less functionally segregated, and more spatially variable: Older children are less spatially homogenous, with the variability of cortical states being related to improved performance on task-based measures (Miskovic et al., 2015). Changes in EEG and very large changes in early ERP components reported in cognitive tasks (Holcomb, Coffey, & Neville, 1992) are in line with the continued changes in brain structure and function that have been observed throughout the adolescent period (Segalowitz, Santesso, & Jetha, 2010). Adolescence thus constitutes the key age that should be investigated relative to the question raised above, namely *when* word production becomes adult-like.

### The Current Study

To fill the gap between previous studies comparing school-age children and adults, here we investigate ERP changes underlying word production in school-age children, adolescents, and adults in a referential word production task. Given that the processing speed is bound to differ across groups, it was necessary to target a specific word-encoding process in the ERP time-course; we therefore manipulated a variable known to similarly affect word production in children and adults, namely AoA of words. Word AoA has been identified as one of the most influential predictors of word production latencies in adults (Chalard, Bonin, Méot, Boyer, & Fayol, 2003; Johnston & Barry, 2006; Juhasz, 2005) and in children (Morrison, Chappell, & Ellis, 1997). It has also been reported as having a solid effect on ERPs in time-windows associated with lexical-phonological encoding in adults (see Perret, Bonin, & Laganaro, 2014; Xue, Liu, Marmolejo-Ramos, & Pei, 2017).

In the present study, we therefore analyze local waveform and global topographic changes associated with picture naming on early- and late-acquired words in four groups of speakers: children (aged 10–12), young adolescents (aged 14–16), older adolescents (aged 17–18), and young adults (aged 20–30). The manipulation of AoA will allow us to follow the onset of the processes modulated by the same psycholinguistics factors across groups. Built on the spatio-temporal segmentation of the signal, the global topographic analyses enable the summary of neural activity in terms of stable electrophysiological activities at scalp (Lehmann & Skrandies, 1984). Combining local waveform and global topographic analyses thus constitutes a great tool to evaluate quantitative (i.e., same topographic patterns, varying in duration) and/or qualitative (i.e., different topographic patterns) differences in neural events across groups of individuals.

## MATERIALS AND METHOD

### Participants

A total of 80 participants divided into 4 groups (children, young adolescents, older adolescents, and adults) participated in the study. Children (*n* = 20; 8 females) were aged from 10 years and zero months to 12 years and 11 months (average: 11;05 ± 0;83). Adolescents were recruited in two groups, with a one-year gap in between used to clearly separate the groups: Young adolescents (*n* = 20; 7 females) were aged from 14 to 16 years (average: 15;26 ± 0;73). Older adolescents (*n* = 20; 12 females) were aged from 17 to 18 years 11 months (average: 17;45 ± 0;51 months). The participants in the adult group (*n* = 20; 13 females) were aged from 20 to 30 (average: 24;45 ± 2;98). All participants were right-handed and French native speakers without diagnosed language impairment or delay or neurological disease. Adults were recruited through announcements posted at the university, whereas children and adolescents were recruited through personal contacts of the authors. This study was approved by the ethical committee of the Faculty of Psychology and Educational Science of the University of Geneva. Written informed consent from adolescents and adult participants, and additional parents’ approval for children and adolescents, were collected, in accordance with the Declaration of Helsinki.

### Tasks and Material

#### Picture naming

The picture naming task used 120 black and white drawings and their corresponding modal names, selected from two French databases (Alario & Ferrand, 1999; Bonin, Peerman, Malardier, Méot, & Chalard, 2003). The stimuli corresponded to words with an AoA range of 1.19–3.55 on a five-point scale (1: *learned between 0 and 3 years*; 4: *learned between 9 and 12 years*; 5: *learned after the age of 12* was not included) to ensure that all the words were known even by the youngest group of participants (see Laganaro et al., 2015) and with high name agreement (mean = 92.5%). The 120 items were split in two groups of 60 items each, corresponding to early-acquired words (mean = 1.73, *SD* = 0.25) and late-acquired words (mean = 2.72, *SD* = 0.59). Early- and late-acquired words were matched on the other relevant psycholinguistic variables from the two mentioned databases (name agreement and visual complexity of the pictures) and cumulative frequency, lexical frequency, number of phonological neighbors, and on four sublexical variables (length in phonemes, sonority values of the first phoneme, syllables, and phonemes frequencies) from the French lexical database Lexique (New, Pallier, Brysbaert, & Ferrand, 2004).

#### Cognitive assessments

In addition to the experimental tasks, the participants underwent several cognitive tests in order to verify that each participant was in the normal range of their respective age and to assess age-related changes in our groups: the vocabulary test and the digit span from the Wechsler Adult Intelligence Scale (Wechsler, 1997) for adolescents from 16 years old and adults, and from the Wechsler Intelligence Scale for Children (Zhu & Weiss, 2005) for the younger participants; two verbal fluency tests (animals and letter P in two minutes) (Cardebat, Doyon, Puel, Goulet, & Joanette, 1990); and a reading fluency test (a short text made up of 265 words). Participants also underwent two reaction time (RT) tests: a simple RT test, consisting in hitting a specific key when a cross appeared on screen, and a choice RT test consisting in having two possible stimuli and two possible responses, in this case hitting “D” on the keyboard when the longer line appearing on the screen was on the left, and “L” when the longest line appeared on the right.

#### Procedure

The participants from the four groups underwent the same picture-naming experiment under EEG/ERP recording in a dimly lit, sound-attenuated room. For the picture-naming task, stimuli were presented on a 17-inch screen (refreshment rate: 50 Hz) at a viewing distance of about 60 cm. The black line drawings appeared on a white foreground, sized at 9.5 × 9.5 cm, against a grey background. The task sequence was controlled by a PC running the E-Prime software (E-Studio). Each trial started with a fixation cross presented for 500 ms in the center of the screen, followed by the appearance of the picture for 3,000 ms, during which the subjects had to overtly produce the word corresponding to the picture as fast and accurately as possible. The interstimulus interval was set at 2,000 ms. All items were presented in two different controlled orders (regular and reversed), with a self-managed break in the middle of the task (after 60 stimuli). The controlled order was preferred to a complete randomization to avoid succession of stimuli from the same semantic category or with high phonological overlap. Four warm-up filler trials were set at the beginning of the experiment and after the break. Overt word productions were recorded by a dynamic microphone, digitally amplified, and the signal was redirected to a computer. Production latencies (RT in milliseconds, i.e. the time separating the onset of the picture and the onset of the speech wave) were systematically checked with speech analysis software (CheckVocal 2.2.6, Protopapas, 2007).

The cognitive assessments were run in a standard room in a face-to-face setting with the experimenter. All the tests were administered in a standard paper-and-pencil setting, except for the two RT tests, which were computer-based.

### Behavioral Data Analyses

For the picture naming task, outliers (RTs shorter than 500 ms or longer than 2,000 ms) and response errors were removed from the dataset. Instances when the answer did not exactly match the expected response, as well as any articles or hesitation marks preceding the answer, were considered errors. RT data were fitted with a linear regression mixed model (Baayen, 2008) and accuracy data were fitted with a generalized linear mixed-effects model for binomially distributed outcomes (Jaeger, 2008) with the R-software (R-project, R Development Core Team, 2005), including participants and items as random effect variables and groups as fixed variable.

For the cognitive assessments, standard scores were calculated when possible (i.e., tasks from the Wechsler assessment), otherwise raw results were reported.

### EEG Acquisition and Preprocessing

The EEG was recorded using the Active-Two Biosemi EEG system (Biosemi V.O.F. Amsterdam, Netherlands) with a 128-electrode cap. Sampling frequency was set at 512 Hz (filters: DC to 104 Hz, 3 dB/octave slope). The custom online reference of the system is the common mode sense active electrode; the driven right leg passive electrode drives the average potentials as close as possible to the amplifier zero (for details on this setup see http://www.biosemi.com). The preprocessing was conducted with Cartool software 3.60 (Brunet, Murray, & Michel, 2011). Epochs of 300 time-frames (TFs) time-locked to 50 TFs before the picture onset (stimulus-locked) and epochs of 250 TFs time-locked to 50 TFs before the vocal onset (response-locked) were extracted and averaged for each subject across conditions. Aligning ERPs to 50 TF (approximately 100 ms) before the vocal onset of each single trial is done to remove prearticulatory motor artifacts (see Fargier, Buerki, Pinet, Alario, & Laganaro, 2018). A minimum of 55 trials was averaged per participant. Data were high-pass filtered at 0.2 Hz and low-pass filtered at 30 Hz (a second order acausal Butterworth filter with −12 dB/octave roll-off) and averaged for each participant and condition (early- or late-acquired words). Stimulus-locked epochs were extracted without baseline correction, and with baseline correction on the 50 prestimulus TFs. Response-locked epochs are without baseline correction, given that there is no consensus on whether and over which period baseline correction should be applied in response-locked ERPs. All epochs related to correct productions were recalculated against the average reference, visually inspected, and accepted only in the absence of artifact, such as eyeblinks, motor artifacts, or large amplitude variations. Only trials with artifact-free stimulus- and response-locked epochs were retained. Contaminated electrodes (max 20% of the 128 electrodes) were interpolated with a 3-D spline interpolation (Perrin, Pernier, Bertrand, Giard, & Echaller, 1987).

### EEG Data Analyses

All analyses were run on average-referenced data. The ERPs were first subjected to a sampling point-wise ERP waveform analysis to determine the time-periods presenting local differences in ERP amplitudes between age-groups and between AoA conditions. Comparisons of each electrode and for each time-point were run separately on stimulus-locked and response-locked ERPs using 4 × 2 analysis of variance (ANOVAs) on amplitudes comparing the four groups in the two conditions using the sten toolbox (https://zenodo.org/record/1167723#.X3IoDmgzaUm). An effect was considered significant if it lasted for at least 10 consecutive TFs and was present on five adjacent electrodes, alpha being set to 0.01.

Because waveform differences do not inform us of the qualitative versus quantitative nature of the observed changes, the main analysis consisted in the spatio-temporal segmentation (topographic or microstate analysis). Considering that periods of stable global electric fields likely correspond to particular periods in mental information processing (Changeux & Michel, 2004; Koukou & Lehmann, 1987; Lehmann & Skrandies, 1984; Lehmann, Strik, Henggeler, Koenig, & Koukkou, 1998), topographic differences across groups would indicate different underlying brain processes. The spatio-temporal segmentation was performed on the group-averaged ERPs of each subgroup and condition, and was statistically validated in the responses of single participants as described below.

Before running the topographic analysis, we performed topographic consistency tests (Koenig & Melie-García, 2010) on stimulus-locked and response-locked ERPs to ensure that different topographies were not driven by random noise in the within-group data. This analysis allowed us to determine that topographic patterns were indeed consistent across subjects (see Supplementary Material in the online supporting information, located at https://www.mitpressjournals.org/doi/suppl/10.1162/nol_a_00024).

The first topographic analysis consisted in identifying periods of significant topographic modulation between groups and conditions on each sampling point on stimulus- and response-locked ERPs. This procedure is called topographic analysis of variance (tANOVA); it consists in a nonparametric randomization test based on global dissimilarities between electric fields, which, in contrast to channel comparisons, computes global dissimilarity of all electric field topographies and tests for the significance of the said topographic differences at each time-point (see an example of its computation in Murray, Brunet, & Michel, 2008). The permutation of the data is accomplished by randomly reassigning the topographic maps of single subjects to the different conditions. The global dissimilarity of these random group-averaged ERPs is compared time-point by time-point with the values of topographic dissimilarity of the actual conditions. The criterion we applied was 12 TFs (about 24 ms) of consecutive significant differences. Neurophysiologically, differences in topography directly indicate changes in the configuration of the active neuronal sources in the brain. In order to test for differences in topography, tANOVA is used. The comparison of the tANOVA result with the microstate segmentation is therefore important to disentangle topographic differences that are yielded by temporal shifts of the same patterns of global distribution of the signal at scalp from topographic differences that are the consequences of different underlying sources.

For the common topographic analysis of stimulus- and response-locked ERPs, the “overlap” between stimulus-locked ERPs and response-locked ERPs was removed based on average RTs of each group, following the procedure described in Laganaro and Perret (2011) and Laganaro (2014). The same procedure was applied to each individual ERP and used in the fitting procedure (see below). As a result, the combination of stimulus- and response-locked ERPs covered the exact time-interval from picture onset to 50 TF before articulation onset for each group and for each participant in each condition.

Scalp electric field topography remains in a stable configuration for a limited period of time: Differences in topography indicate changes in the configuration of the active neuronal sources in the brain (Murray et al., 2008). This analysis involves a spatio-temporal segmentation of the ERPs over periods of electrophysiological stability, by compressing the variability of ERPs in a series of topographical maps that summarize data and indicate which template explains ERPs in each group. A temporal atomized and agglomerate hierarchical clustering algorithm (Murray et al., 2008) to identify the template maps was applied to the group-averaged data. With the aim of selecting the optimal number of maps most likely explaining the ERP signal, the minimum remaining time of a given topography was 12 TFs (approximately 24 ms) with 95% correlation. Microstate segmentation was applied to the eight group- and condition-averaged ERP data sets. To determine whether the differences observed on the grand averages reflect a single participant’s neural responses, a comparison between grand averages and individual data was conducted. This “fitting” procedure, provides the information required to understand how much a topographic map observed in a given group actually explains an individual participant’s ERP data. Measures of map (microstate) presence and global explained variance (GEV) in each individual ERP were obtained and used to test statistical differences among groups and conditions. When a topographic map was consistently present, its duration in the participants’ ERP signal was also used to compare conditions.

## RESULTS

### Behavioral Results

The performances of all the participants were within the norms on the vocabulary test and the digit span memory test. On the other cognitive tasks, where no standard score was available, no subject obtained outlying results. On the three tests where a comparison was possible (due to age adaptation, some of the tests were not identical, see Materials and Method), an increase of performance can be observed across the age groups. Table 1 displays the results per group on these cognitive tests. In both verbal fluencies (animals and letter), a group effect was observed—respectively, Kruskal-Wallis test *H*(3, *N* = 80) = 14.80443, *p* = 0.0020, and Kruskal-Wallis test *H*(3, *N* = 80) = 21.73078, *p* = 0.0001—with children providing fewer items per category than older adolescents and adults (animals: *z* = 3.54, *p* = 0.002, and *z* = 3.06, *p* = 0.01; letter: *z* = 3.65, *p* = 0.001 and *z* = 4.32, *p* = 0.00009), but without significant difference with young adolescents (animals: *z* = 2.27, *p* = 0.15; letter: *z* = 2.64, *p* > 0.05). A group effect was also observed on reading fluency—Kruskal-Wallis test *H*(3, 79) = 18.55257, *p* = 0.0003—with slower reading times in children relative to older adolescents and adults (*z* = 3.893, *p* = 0.0006, and *z* = 3.335, *p* = 0.005). Young adolescents did not differ from the other groups. Similar results appeared on simple and choice RT—group effect simple: Kruskal-Wallis test: *H*(3, *N* = 80) = 14.76358, *p* = 0.002; group effect choice: Kruskal-Wallis test: *H*(3, *N* = 80) = 21.09, *p* = 0.0001. Older adolescents and adults differed from children, who displayed the slowest processing speed (simple older adolescents: *z* = 3.368, *p* = 0.005; simple adults: *z* = 3.035, *p* = 0.01; choice older adolescents: *z* = 4.062, *p* = 0.0003; choice adults: *z* = 3.538, *p* = 0.002), whereas young adolescents did not differ from children, nor from older adolescents, nor from adults.

**Table 1.** Mean results on the cognitive tests for each age group

	Verbal fluency (score)		Reading fluency (seconds)	Reaction time (ms)	
	Category	Letter		Simple	Choice
Children	28	19	123	325	469
Young adolescents	35	25	87	315	418
Older adolescents	40	28	78	290	369
Adults	37	28	75	278	358

*Note*. Each score represents the number of correct answers provided by the group.

**Figure 1.**
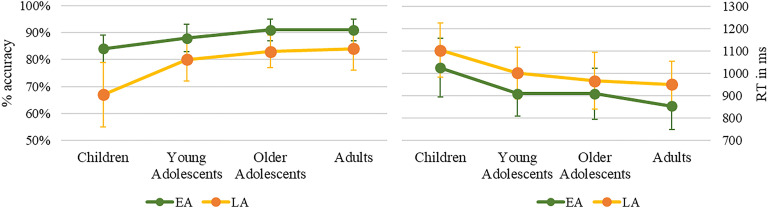
Naming accuracy (in %) and reaction times (RT; in ms) for groups and conditions. EA: early-acquired words, LA: late-acquired words.

In the picture-naming task (see Figure 1), production accuracy was lower in children relative to adults (*z* = −4.508, *p* < 0.0001, β = −0.83, *SE* = 0.184) and in young adolescents relative to adults (*z* = −2.133, *p* < 0.05, β = −0.405, *SE* = 0.1897), but without significant difference between the two groups of adolescents (*z* = −0.364, *p* = 0.716, β = −0.0714, *SE* = 0.19611), both differing from children (young adolescents: *z* = −4.588, *p* < 0.0001, β = −1.104, *SE* = 0.24; older adolescents: *z* = −6.092, *p* < 0.0001, β = −1.488, *SE* = 0.24). Furthermore, accuracy was better for early-acquired words than for late-acquired words, in all groups (children: *z* = 4.905, *p* < 0.0001, β = 1.4598, *SE* = 0.2976; young adolescents: *z* = 2.533, *p* < 0.05, β = 0.7774, *SE* = 0.30687; older adolescents: *z* = 2.306, *p* < 0.02, β = 0.725, *SE* = 0.314; adults: *z* = −1.969, *p* < 0.05, β = −0.625, *SE* = 0.3172).

Production latencies also differed across groups, *F*(3, 79.8) = 10.035, *p* < 0.0001, and word AoA *F*(1, 118.8) = 23.2385, *p* < 0.0001, without interaction between groups and AoA, *F*(3, 7829.3) < 1. Between-group differences were significant for children as compared to both adolescent groups, young adolescents: *t*(86.4477) = 3.298, *p* = 0.001; older adolescents: *t*(86.2356) = 4.337, *p* < 0.0001, and to adults, *t*(79.985) = 5.075, *p* < 0.0001. Both adolescent groups did not differ from adults, young adolescents: *t*(79.674) = 1.666, *p* = 0.0996; older adolescents: *t*(79.498) = 0.972, *p* = 0.334, nor among them, *t*(85.02) = −1.04, *p* = 0.3.

### ERP Results

#### Waveforms

**Figure 2.**
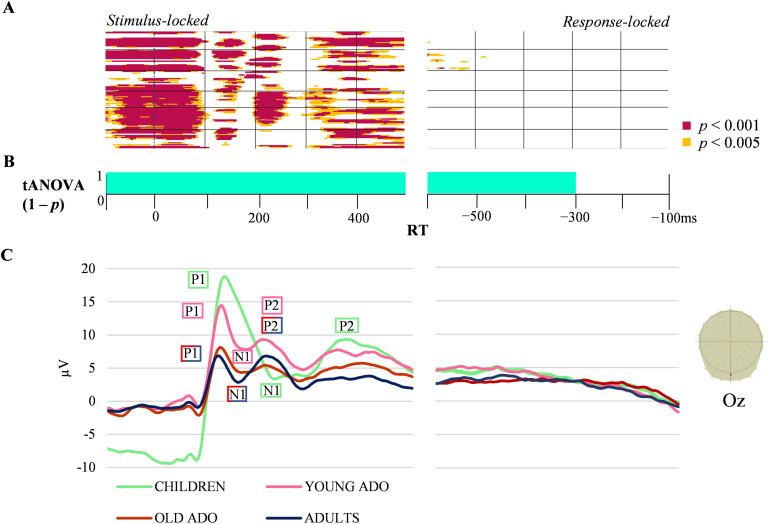
Children, young & older adolescents and adults group-averaged stimulus-aligned and response-aligned event-related potentials (ERPs; 128 electrodes). (A) Periods of significant differences in amplitudes across groups on each electrode and time-frame for the stimulus-locked and response-locked ERPs. (B) Periods of significant differences in the tANOVA analysis (*p* < 0.01) in turquoise. (C) Exemplars of group-averaged ERP waveforms (Oz) for the four groups are plotted in microvolts in function of time (bottom panel). The shifting of the components in the stimulus-locked ERPs is labeled (with older adolescents (ADO) and adults sharing the label due to similar amplitudes and components). RT: reaction time.

**Figure 3.**
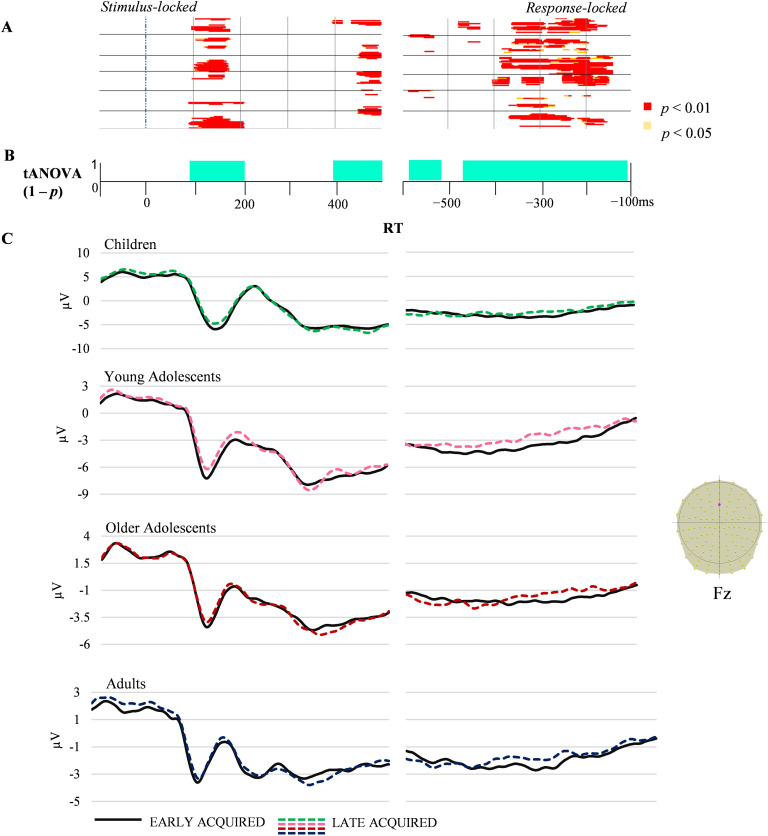
Condition-averaged stimulus- and response-locked event-related potentials (ERPs; 128 electrodes). (A) Periods of significant differences in amplitudes between early- and late-acquired words on each electrode and time-frame for the stimulus-locked and response-locked ERPs. (B) Periods of significant differences in the tANOVA analysis (*p* < 0.01) are displayed in turquoise. (C) Exemplar condition-averaged ERP waveform (Fz) for the four groups plotted in microvolts in function of time. RT: reaction time.

The 4 × 2 ANOVA showed significant time-periods of group effect (Figure 2; see Supplementary Materials in the online supporting information for stimulus-locked periods of differences in amplitudes and exemplar of the group-averaged waveform [Oz] for the four groups) and also of AoA effect (Figure 3) on ERP waveforms, without interaction between groups and AoA condition. The group effect yielded significant differences in amplitudes on most electrodes throughout the entire stimulus-locked time-period, whereas virtually no significant differences were found in the response-locked ERPs (Figure 2). The extended statistical differences in time and space in the stimulus-locked ERPs reflect the larger amplitudes and latency shifts between the groups observed on the exemplar of waveforms in Figure 3. The younger groups were characterized by higher amplitudes and delayed latencies of ERP components. The results of waveform analyses computed on two consecutive groups at a time (see Appendix A in the online supporting information) indicated that amplitudes differ between children and young adolescents in the very first time-window (−100 to 100 ms) and from about 190 to 250 ms. Between young and older adolescents, amplitudes were significantly different only on a short time-window around 110 ms, but waveform amplitude differed between young adolescents and adults in the 300 to 350 ms time-window on a large proportion of electrodes.

To validate the visually-observed latency shift, the latency of the P1 component (around 100 ms post picture onset) was also extracted for each age group based on the latency of maximal global field power value in the 80 to 160 ms time-window. A group effect was observed, Kruskal-Wallis test *H*(3, 160) = 20.74, *p* = 0.0001, with a later P1 peak in children relative to older adolescents and to adults (*z* = 3.96, *p* = 0.0004 and *z* = 3.84, *p* = 0.0007), but without significant difference with young adolescents (*z* = 2.13, *p* = 0.2). Given these results, we computed the correlation between the P1 peak and age. On the entire group of participants, the negative correlation was low although significant (*r* = −0.21, *p* < 0.05), whereas on the two youngest groups the correlation was not significant (*r* = −0.14, *p >* 0.05).

The AoA effect yielded different amplitudes between early- and late-acquired words around 100 ms after stimulus appearance and towards the end of the stimulus-locked signal, after 400 ms, on most electrodes (Figure 3). On the response-locked ERPs, a long period covering from 480 to 100 ms prior to participant response showed significant differences on most electrodes, and a shorter significance period of 80 ms starting around 600 ms before articulation was observed on central bilateral electrodes (Figure 3).

#### tANOVA

The tANOVA analysis also revealed differences across groups throughout all the stimulus-locked ERPs, but also 600 to 300 ms prior to the vocal onset for the response-locked ERPs (see Figure 2). For AoA, the tANOVA was significant in the same time-windows described for amplitudes.

#### Microstate analysis

**Figure 4.**
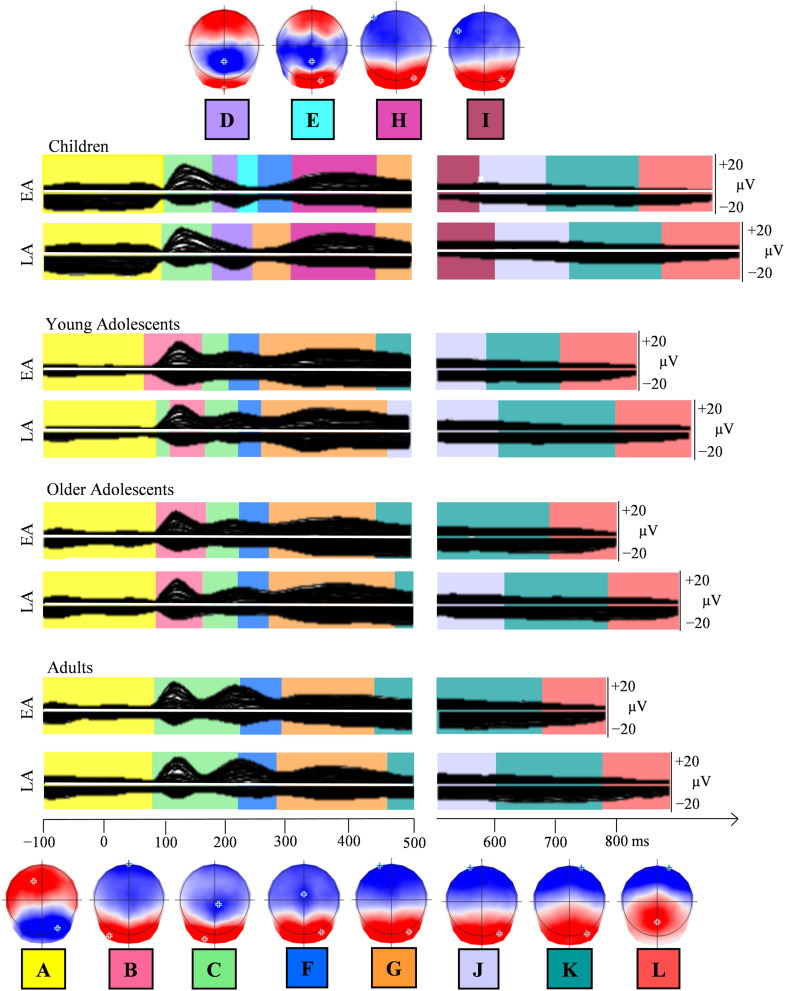
Group-averaged event-related potentials (ERPs; 128 electrodes) for each group (children, young and older adolescents, adults) and condition (EA & LA) from 100 ms before the stimulus onset to 100 ms before vocal onset (after the concatenation process, which removed the overlap between stimulus-locked and response-locked ERPs according to each group average response latencies). The temporal distribution of the topographic maps in each data set revealed by the spatio-temporal segmentation analysis is superposed with code colors. The 12 corresponding template maps are displayed with positive values in red and negative values in blue. EA: early-acquired words, LA: late-acquired words.

The microstate segmentation applied on the eight grand averages (4 groups × 2 conditions) revealed a best model explaining 95% of variance with 12 different topographic maps from 100 ms before picture onset to 100 ms before vocal onset (Figure 4). On the grand averages, different periods of topographic stability appeared for children, as compared to older groups, in early time-windows (maps D, E, H in Figure 4). Both groups of adolescents displayed similar maps to adults, after the P1 component. On response-locked epochs, the sequence of topographic maps was similar across age-groups.

Three fitting periods were applied to the individual combined stimulus- and response-locked ERPs to statistically assess the differences observed on the grand averages. The first fitting period was meant to target the microstate underlying the P1 component, thus spanned from 35 TF to 103 TF (68 to 202 ms) after picture onset, testing for Maps B and C. The second period spanned from 100 TF (~200 ms) to the end of the stimulus-locked signal and included maps D, E, F, G, and H. In the third period covering the remaining individual ERPs locked to response onset, maps I, J, K, and L were fitted. The results of the fitting in the individual ERPs, in terms of presence, global explained variance, and duration, are displayed in Appendix B in the online supporting information.

##### Group comparisons.

Among the two microstates observed around the P1 component, Map B was present in most participants from the three older groups and in only half of the children, whereas Map C was observed in 19 out of 20 children but also in a high proportion of participants from the other groups (see Presence in Appendix B in the online supporting information). The different distribution of the two P1 maps across groups was statistically confirmed by the interaction between groups and maps on the GEV, *F*(3, 76) = 6.07, *p* = 0.009. Pearson’s correlation computed between age and GEV of Map B on all participants showed no significant correlation (*r* = −0.14, *p* > 0.05), whereas a significant negative correlation was observed on Map C (*r* = −0.49, *p* < 0.05), further showing that the presence of Map C decreases with age.

In the second fitting period, Map D and—to a lesser extent—Map H are predominantly present in children as opposed to all other groups (see Presence in Appendix B in the online supporting information). This was confirmed by the statistical analyses, with children differing from all the other groups (*p* < 0.05) on GEV, D: *F*(3, 76) = 7.846, *p* = 0.0001, *MSE* = 0.0004; *H*: *F*(3, 76) = 7.318, *p* = 0.001, *MSE* = 0.015, except for the comparison with young adolescents on Map H, where there is no significant difference. There was actually a significant correlation between GEV and age on these two groups (children and young adolescents: *r* = −0.44, *p* < 0.05), showing that the presence of Map H decreases between the ages of 10 and 16. Presence in the individual ERP signal was very low for Map E and will not be analyzed further. Map F was present in about half of the participants from each group (see Presence in Appendix B in the online supporting information) without statistical difference across groups.

Map G was consistently observed in all the groups, but with between-group differences in the explained variance, *F*(3, 76) = 5.958, *p* = 0.001, *MSE* = 0.048, and on duration, *F*(3, 76) = 6.4735, *p* = 0.0005, *MSE* = 19,174, with children differing from all the other groups on GEV (*p* < 0.05) and young adolescents differing from older adolescents and adults (*p* < 0.05). Map G actually better characterized the ERP signal in the young adolescent group relative to the other groups (see GEV and Duration in Appendix B in the online supporting information).

Maps K and L were present in the ERP signal of the participants from all groups. This was less the case for Maps I and J, which were more present in children and adolescents than in adults. In fact, the GEV for Map J yielded a group effect, *F*(3, 76) = 3.21, *p* = 0.02, *MSE* = 0.006, with young adolescents differing from adults. Map K did not present any significant effect on either GEV or duration. Map L presented a group effect on GEV, *F*(3, 76) = 2.87, *p* < 0.05, *MSE* = 0.003, with children differing only from adults.

##### Age of Acquisition comparisons.

In terms of AoA, there was a significant effect on the P1 Maps, *F*(1, 76) = 25.20, *p* < 0.0001, with higher explained variance for early-acquired words, and no interaction between AoA and Maps or between AoA and groups. Map D, which was specific to children, was modulated by AoA on GEV, *F*(1, 76) = 9.8586, *p* = 0.003, *MSE* = 0.00001, with higher GEV for late-acquired words. AoA modulated both GEV and duration on Map G, GEV: *F*(1, 76) = 5.958, *p* = 0.04, *MSE* = 0.048; duration: *F*(1, 76) = 6.8975, *p* = 0.01, *MSE* = 2,690, with slightly longer lasting and higher GEV for early-acquired words (8 ms). No interaction was observed between AoA and groups. On Map I an AoA effect was observed on GEV, *F*(1, 76) = 5.134, *p* = 0.02, *MSE* = 0.0006, with late-acquired words presenting a larger GEV, but no group effect or interaction. Map L yielded an AoA effect on duration, *F*(1, 76) = 16.78, *p* = 0.0001, *MSE* = 13,708, with longer duration for late-acquired words.

To summarize, behavioral results indicated that only children differed from all other groups in picture-naming speed. On accuracy and on the other cognitive tasks, children statistically differed only from the two older groups, with young adolescents being somehow intermediate. In the ERP waveform analyses, young adolescents displayed intermediate amplitudes between children and older adolescents and adults on the stimulus-locked ERPs. Different patterns underlying the P1 component were observed in children (Map C) relative to other groups (Map B). Different time distributions of a specific pattern underlying the P2 component (see Figure 3C) were observed in children (Map H), and Map G differed in adults relative to the older group of adolescents. Age of acquisition effects were observed on topographies approximately in the same time-windows as amplitude differences in all groups, namely around the P1 component and after 400 ms.

## DISCUSSION

The aim of the present study was to investigate ERP differences in single-word production in two groups of adolescents compared to children and adults. In particular, we intended to determine when and how the behavioral and neurophysiological processes underlying word production in a picture-naming task become adult-like. Reaction times were significantly slower only in children as compared to adolescents and adults. Both adolescent groups did not differ significantly from adults, suggesting an already adult-like behavioral outcome from 14-year-old adolescents. In terms of accuracy of responses, children as well as the younger group of adolescents (14 to 16 years old) were less precise than adults, whereas the older group of adolescents (17 to 18 years old) did not differ from adults. The same results were also observed on the other cognitive tasks (verbal fluency, RTs, reading fluency), where the group of young adolescents did not differ significantly either from children or from older adolescents and adults.

At the neurophysiological level, waveform amplitudes clearly decreased across the first three age-groups, with the young adolescent group being intermediate between children and the two other groups. Also, children displayed different global field potentials in early time-windows (P1-N1), all other groups being alike in these time-windows. However, young adolescents also yielded some differences relative to the two older groups on some topographies in the stimulus-locked data, in particular around 300–400 ms after picture onset. No group differences appeared in the response-locked data on amplitudes, and an extremely similar sequence of stable global electric fields at scalp was observed in the four groups, with only different time distribution.

A first interpretation of these results is that brain activity underlying referential word production is completely adult-like in the adolescents over 17 years old, whereas a somehow intermediate pattern is still observed in the group aged 14 to 16 years old. In the following we will discuss changes across groups, first on amplitudes, then on microstates, and integrate the functional interpretation in terms of word-planning processes by integrating the results of the manipulated psycholinguistic factor (word AoA).

### Amplitude and Latency Decrease from Childhood to Adolescence

The present results indicate larger amplitudes in children and young adolescents compared to adults and older adolescents, whose signal is equivalent. Amplitudes and latency of the P1 component are intermediate between children and adults in the 14- to 16-year-old adolescents and are adult-like after the age of 17. Such differences in amplitudes are observed only in the stimulus-locked ERPs.

Developmental changes, especially in the P1 time-range of the visual evoked potentials (VEP; a positive ERP component on posterior electrodes, occurring approximately 100 ms after stimulus presentation) have been well documented in the literature. Amplitudes decrease with age, and have been linked to the progressive thickening of children’s skulls (Chauveau et al., 2004; Picton & Taylor, 2007), as well as to the growing age-related automaticity in the processes required by the task (Durston & Casey, 2006), automatization being a result of the combination of myelination (Picton & Taylor, 2007) and synaptic pruning (Itier & Taylor, 2004; Taylor, Edmonds, McCarthy, & Allison, 2001). The latency shift of the P1 component also decreases with maturation, most likely reflecting the acceleration of stimulus processing (Itier & Taylor, 2004; Taylor et al., 2001). Moreover, some studies (Hopfinger & Mangun, 1998; Luck, Woodman, & Vogel, 2000) agree on the fact that attention has an effect on modulating visual-spatial cortical sensory processing, which results in shifted latencies of the P1 among the youngest participants. Developmental changes have also been reported in the N1-N170 time-window, with amplitudes becoming more negative with age and the latency decreasing in visual discrimination tasks (Batty & Taylor, 2006; Itier & Taylor, 2004). N1 is another major VEP component, characterized by a central negative peak around 170 milliseconds after stimulus presentation (Creel, 2019). Similarly to our findings, previous VEP studies showed that children have different ERPs relative to adolescents and adults. In particular, an additional component is observed in children’s ERPs in the N170-like time-window (Campbell & Sharma, 2016; Yadav, Poudel, Limbu, Thakur, & Yadav, 2015). Here, the period of electrophysiological stability observed in this time-window (see topography of map D, Figure 4) has previously been reported for N1 (N170-like) adult ERPs (Fargier & Laganaro, 2016; Laganaro, 2017; Rossion & Caharel, 2011); we therefore interpreted the stability as a single N170-like component.

The stabilization of the VEP component latency is specified to occur only around the twentieth year of age (Allison, Hume, Wood, & Goff, 1984; Emmerson-Hanover, Shearer, Creel, & Dustman, 1994; Shaw & Cant, 1981). In our data, this seems to occur earlier, because the P1 peak appears already adult-like in the older adolescent group. Nonetheless, other studies affirm not showing reliable change across young age (Mahajan & McArthur, 2012; Snyder, Dustman, & Shearer, 1981). Here, amplitude decreases also concern the P2 component, which will be further discussed in relationship with the corresponding microstate.

In sum, the ongoing amplitude shrinking is coherent with the literature although stabilization seems to occur earlier, which might be linked to stimulus processing, thus not observed in the response-locked amplitudes.

### Microstates

As for amplitudes, topographic differences across groups are observed only in early time-windows. The first topographic differences observed on the averaged ERPs appeared in the P1 time-window. In this time-window, the topographic map that was typically specific to the children’s signal (Map C in Figure 4) showed decreased presence with age, and young adolescents differed from the rest of the groups because they displayed the most consistent presence of the microstate corresponding to Map B in Figure 4. The presence of a different P1 microstate in children as compared to adults has been reported previously (Holcomb et al., 1992; Laganaro et al., 2015; Shaywitz et al., 2007) and has been attributed to more bilateral occipital activation in children.

Microstates also differed across groups in the N1-like time-window. Here again, children are the only group statistically different from the other 3 groups (Map D and E). Children are actually the only group in which an N1 map is consistently present (Maps D and E), although it is temporally shifted by the delay of the P1 latency described in the previous section. Interindividual inconsistency in the N1 time-window in picture-naming tasks has been previously reported in (young) adults (see for instance Laganaro, 2017, on a large group of participants). Here, the results show that such inconsistency is already present in the young adolescent group (i.e., from the age of 14). The N1/N170 component has been associated with conceptual processes for pictorial stimuli (Schendan & Kutas, 2003). It has been shown to be modulated by the category of the picture (Thorpe, 2009), and this modulation could be due, among other factors, to the expertise level of subjects for a given category (Taylor, Batty, & Itier, 2004). The present results suggest that picture-to-concept processes are different in children only and are already adult-like in younger adolescents. Such modulation across ages may reflect either changes in visual-conceptual processes, which are not specific to picture naming, or visuo-conceptual activation that specifically guides lexical selection.

The P2 peak, the second component most likely underlying Levelt’s (2001) concept-to-lexical processes (selection of the lemma), confirmed by ERP studies on picture naming (Aristei, Melinger, & Abdel Rahman, 2011; Laganaro et al., 2015; Laganaro, Valente, & Perret, 2012; Maess, Friederici, Damian, Meyer, & Levelt, 2002; Strijkers et al., 2010), also showed differences between children and the older groups, in terms of latencies and underlying topographies (Map H in children and G in the other groups). Whereas different latencies in children can be the consequence of previous shifts (P1 and N1), there is a significant change in the underlying microstate already between children and young adolescents, whose ERP signal is better explained by the same map that is in the other older groups. The present results in children differ from previous results in Laganaro et al. (2015), where the same P2 Map was observed in children and adults. The two P2 topographies (Maps G and H) are however very close and highly correlated (95%). The younger adolescents display the adult P2—Map G. Actually, as already observed on the P1 map, the P2 “adult” map is also more consistent in young adolescents than in older adolescents and in adults. In general, along the whole signal, young adolescents seem indeed to present considerably different topographies from older adolescents and adults, although their amplitudes remain intermediate.

No significant between-group differences are observed beyond the P2 map in terms of presence. There are hence only differences in duration on the rest of the microstates, which will be discussed in further detail in relationship with the AoA effect.

### Dynamics of Word Planning

The manipulation of the AoA was aimed at pinpointing lexical-phonological processes (Perret et al., 2014), and thus, at interpreting the time-course of different encoding processes from childhood to adolescence and to adulthood. Amplitudes and spatio-temporal analyses converged in identifying that AoA consistently modulated ERPs after 430 ms and mainly in the response-locked ERPs, except for an auxiliary time-window around the P1 component. Crucially, there was no interaction with groups, showing that AoA affects the same time-windows at all ages. While the P1 modulation is very likely due to different picture properties across early- and late-acquired words (see Perret & Laganaro, 2012, for similar results), the later AoA effect likely reflects the time-window of word-form encoding. The large time-window modulated by AoA also corresponds to the period in which no significant differences appeared on ERPs across groups on either amplitudes or topographies.

As highlighted above, qualitative differences between groups appear mainly for children in the P1-N1-P2 time-window, whereas the corresponding microstates are already adult-like in the signal of the younger adolescents. On the other hand, amplitudes are still intermediate between children and adults in the young adolescents in these time-windows. According to the results of the AoA effect, as well as to previous estimates on the time-course of word production in picture naming (Indefrey, 2011), these early time-windows correspond to prelexical (visual and conceptual; P1 component), and to word (lemma) retrieval (P2 component). These results suggest that brain processes are the same for all groups for lexical and postlexical word-encoding processes and that visual-to-conceptual, and possibly conceptual-to-lexical, encoding differs only in children before the age of 14, at least in the sequential approaches to word planning processes. A different interpretation would stem in the framework of theories claiming a parallel activation of lexical and phonological information (Miozzo et al., 2014; Strijkers et al., 2017). In such accounts, lexical-semantic and phonological-articulatory processes emerge together rapidly (i.e., the firing hypothesis), drawing in parallel on temporal and frontal cortex around 150–200 ms after the presentation of the picture. In this interpretation, the differences between groups may reflect different patterns of information flow between children and adults, either serial versus parallel processes or different degrees of overlap between serial processes.

The intermediate amplitudes in these same time-windows in the 14 to 16 year olds reflect either the ongoing brain maturation as discussed above or an increased recruitment of the same network as in adults, to deliver the same performance on the same task. Visually mediated processes around 100 ms after stimulus onset, and supposedly lexical-semantic selection processes around 300 ms, are then undergoing changes. These brain processes do not seem to develop at the same pace (visual processes being already wired among young adolescents before other later-developing processes, possibly related to lexical-semantic encoding), and they seem to recruit different brain regions. The middle temporal gyrus, and partly, the inferior frontal gyrus, are allegedly involved in the lexical-semantic processes (Indefrey & Levelt, 2004). The developmental curve of these two regions is not analogous, with the cortical gray matter of the temporal cortex peaking at 16 years old (Giedd et al., 1999; Lenroot & Giedd, 2006), which may explain the ongoing maturation. In fact, when looking back at the question of *when* children become adult-like, there is no strong assumption that all processes should mature at once, but rather they should be gradual because different processes involve different brain regions with some processes becoming adult-like before others.

### Conclusions

**Figure 5.**
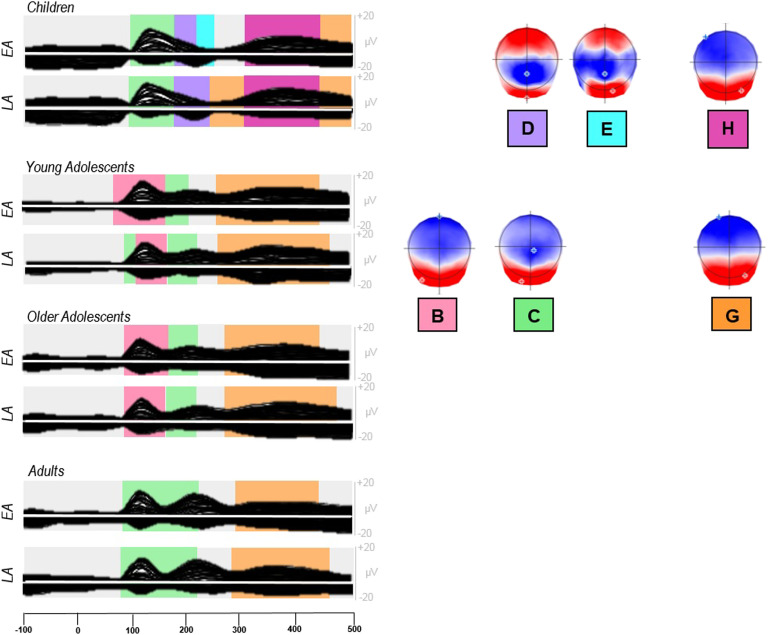
Group-averaged event-related potentials for each group (children, young adolescents, older adolescents, adults) and condition (EA & LA) isolating and only showing the topographical maps of interest. EA: early-acquired words, LA: late-acquired words.

The present study analyzed the waveform amplitudes and the periods of stable electric fields (topographies) in the ERP signal from picture onset to the individual onset of articulation in children, adolescents, and adults. This allowed us to track simultaneously the behavioral, functional, and temporal changes in the development of the word-encoding processes. Our results show that children are outliers compared to the older participants in all measures (in terms of naming latency and accuracy, and in ERP amplitudes, topography, and morphology; see Figure 2 and Figure 5). Moreover, adolescents from the age of 17 years old seem to have reached, not only behaviorally, but also temporally and functionally, adult-like brain activation. Young adolescents (aged 14 to 16), although being closer to adults than to children in terms of processing speed and microstates, still display intermediate results on accuracy and ERP amplitudes. An important shift in brain signals and in referential word production processing speed seems thus to occur between the ages of 12 and 14. This period should therefore be further investigated with improved granularity and on additional word production tasks.

## ACKNOWLEDGMENTS

This work was supported by Swiss National Science Foundation (SNSF) grant no. 100014_165647.

## FUNDING INFORMATION

Marina Laganaro, Schweizerischer Nationalfonds zur Förderung der Wissenschaftlichen Forschung (http://dx.doi.org/10.13039/501100001711), Award ID: 100014_165647.

## AUTHOR CONTRIBUTIONS

Tanja Atanasova: Conceptualization, Methodology, Validation, Data curation, Formal analysis, Investigation, Writing – Original draft, Visualization. Raphaël Fargier: Conceptualization, Methodology, Writing – Review & editing. Pascal Zesiger: Conceptualization, Methodology, Writing – Review & editing, Funding acquisition. Marina Laganaro: Conceptualization, Methodology, Validation, Resources, Writing – Review & editing, Supervision, Project administration, Funding acquisition.
