# Supplementary material for: Dynamics of Word Production in the Transition from Adolescence to Adulthood
Source: Neurobiol Lang (Camb). 2020 Nov 1;2(1):1–21. doi: 10.1162/nol_a_00024 (PMC10158562; doi:10.1162/nol_a_00024)
Supplement: Supplementary file 1 [file nol-2-1-1-s001.pdf]

Supplementary Material

1. TCT Stimulus-locked

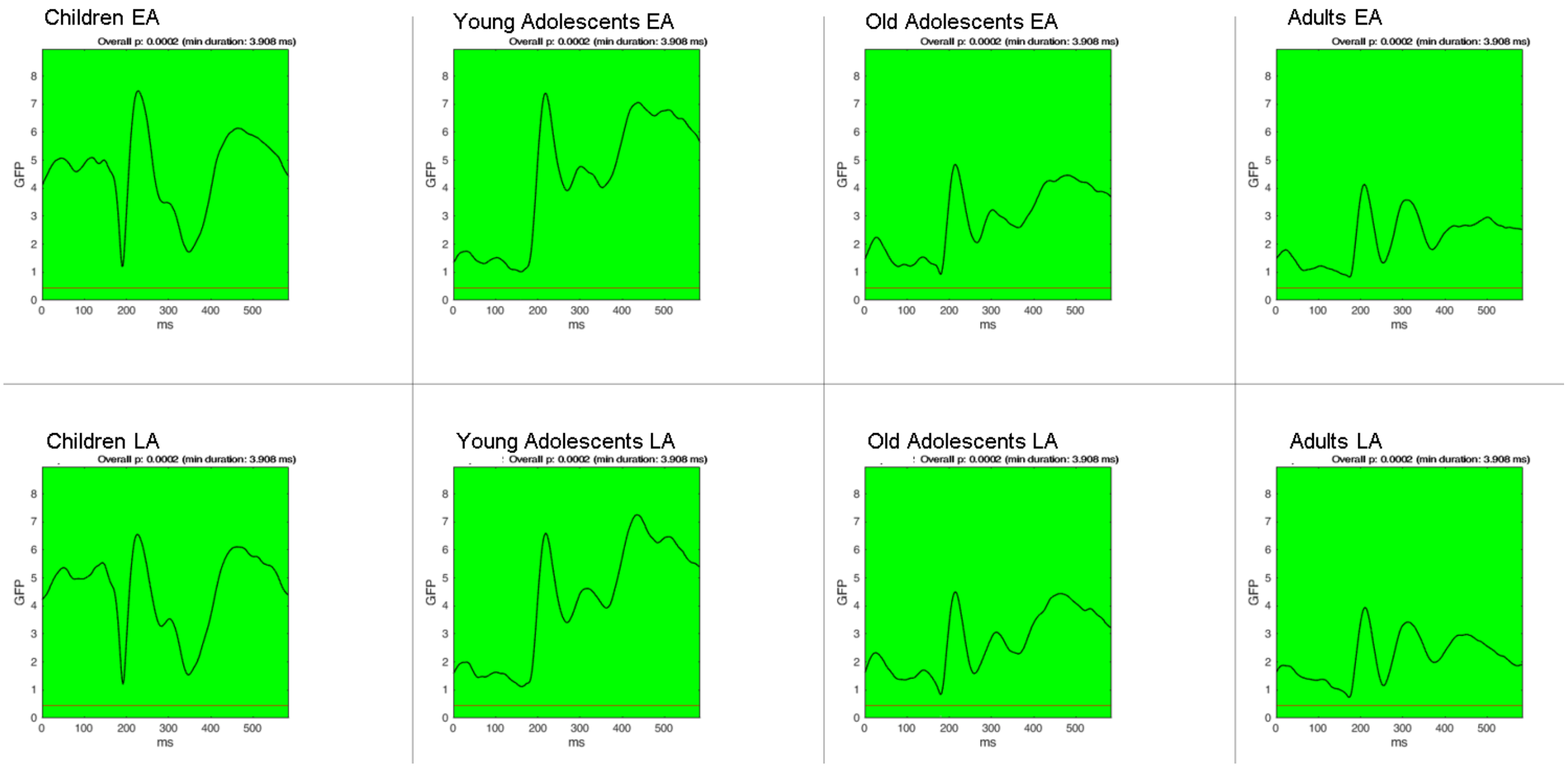

2. TCT Response-locked

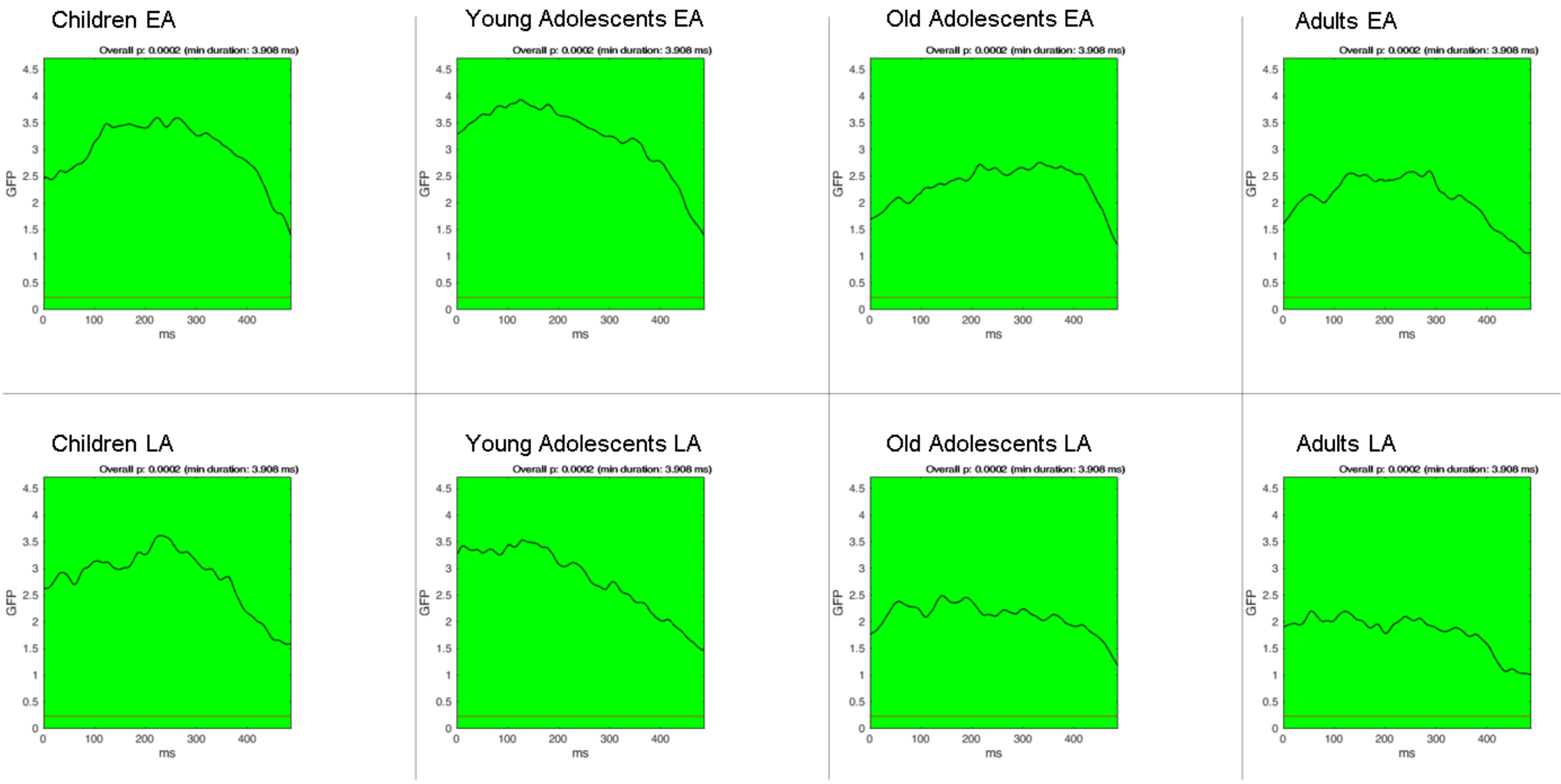

3. Baseline-corrected analyses and waveform

A

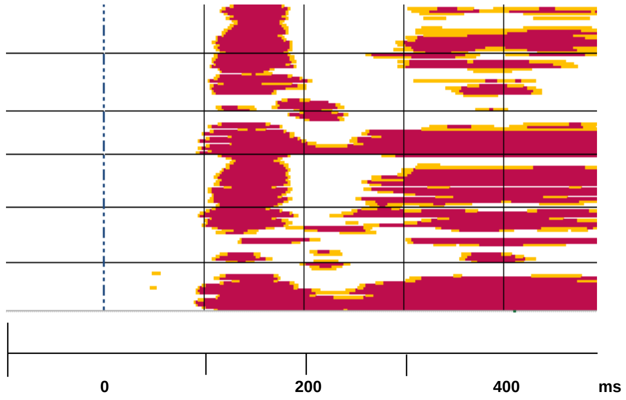

B

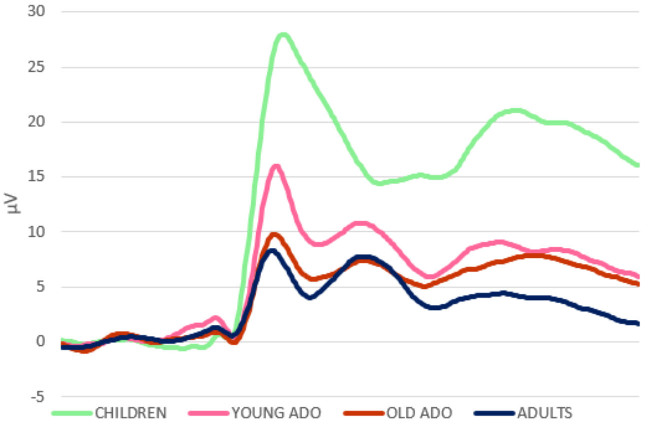

Figure S1. A. Periods of significant differences in amplitudes across groups on each electrode and time-frame on the stimulus-locked ERPs with baseline correction. B. Exemplar of group-averaged baseline corrected stimulus-locked ERP waveforms (Oz) for each group are plotted in microvolts in function of time.
